# Acute Pharmacological Effects of 2C-B in Humans: An Observational Study

**DOI:** 10.3389/fphar.2018.00206

**Published:** 2018-03-13

**Authors:** Esther Papaseit, Magí Farré, Clara Pérez-Mañá, Marta Torrens, Mireia Ventura, Mitona Pujadas, Rafael de la Torre, Débora González

**Affiliations:** ^1^Clinical Pharmacology Unit, Germans Trias i Pujol University Hospital, Institute for Health Science Research Germans Trias i Pujol, Badalona, Spain; ^2^Department of Pharmacology, Therapeutics and Toxicology and Department of Psychiatry and Forensic Medicine, Autonomous University of Barcelona, Barcelona, Spain; ^3^Drug Addiction Program, Institute of Neuropsychiatry and Addictions, Barcelona, Spain; ^4^Energy Control, Associació Benestar i Desenvolupament, Barcelona, Spain; ^5^Integrative Pharmacology and Systems Neuroscience Research Group, Neurosciences Research Program, Hospital del Mar Medical Research Institute, Barcelona, Spain; ^6^Department of Experimental and Health Sciences, Pompeu Fabra University, Barcelona, Spain

**Keywords:** 2C-B (2, 5-dimethoxy-4-bromophenethylamine), psychedelic, phenylethylamines, psychostimulants, cortisol

## Abstract

2,5-dimethoxy-4-bromophenethylamine (2C-B) is a psychedelic phenylethylamine derivative, structurally similar to mescaline. It is a serotonin 5-hydroxytryptamine-2A (5-HT_2A_), 5-hydroxytryptamine-2B (5-HT_2B_), and 5-hydroxytryptamine-2C (5-HT_2C_) receptor partial agonist used recreationally as a new psychoactive substance. It has been reported that 2C-B induces mild psychedelic effects, although its acute pharmacological effects and pharmacokinetics have not yet been fully studied in humans. An observational study was conducted to assess the acute subjective and physiological effects, as well as pharmacokinetics of 2C-B. Sixteen healthy, experienced drug users self-administered an oral dose of 2C-B (10, 15, or 20 mg). Vital signs (blood pressure and heart rate) were measured at baseline 1, 2, 3, 4, and 6 hours (h). Each participant completed subjective effects using three rating scales: the visual analog scale (VAS), the Addiction Research Centre Inventory (ARCI), and the Evaluation of the Subjective Effects of Substances with Abuse Potential (VESSPA-SSE) at baseline, 2–3 and 6 h after self-administration (maximum effects along 6 h), and the Hallucinogenic Rating Scale (maximum effects along 6 h). Oral fluid (saliva) was collected to assess 2C-B and cortisol concentrations during 24 h. Acute administration of 2C-B increased blood pressure and heart rate. Scores of scales related to euphoria increased (high, liking, and stimulated), and changes in perceptions (distances, colors, shapes, and lights) and different body feelings/surrounding were produced. Mild hallucinating effects were described in five subjects. Maximum concentrations of 2C-B and cortisol were reached at 1 and 3 h after self-administration, respectively. Oral 2C-B at recreational doses induces a constellation of psychedelic/psychostimulant-like effects similar to those associated with serotonin-acting drugs.

## Introduction

Psychedelics have been traditionally classified by either their chemical structure or primary mechanism of action into two classes: serotonergic hallucinogens (indolamines, e.g., psilocybin and LSD) and phenylethylamines [e.g., mescaline and 2,5-dimethoxy-4-iodoamphetamine (DOI)] (Vollenweider, 2001; Aarde and Taffe, 2017). Recently, however, new psychoactive substances (NPSs) developed from both substitutions and well-known structures have emerged.

Such novel psychedelics include the 2C-series and its structural analogs, including *N*-Benzylphenethylamines (NBOMes) (Tracy et al., 2017). 2C-series, also called 2C-drugs/compounds, are a related group of substances with presumably psychedelic and psychostimulant properties. All of them are phenylethylamine derivatives structurally close to mescaline with methoxy substitutions at the 2 and 5 positions derived from the two carbon molecules between the benzene ring and the amino group. 2,5-dimethoxy-4-bromophenethylamine (2C-B, Nexus) is one of the oldest and best known 2C-type drugs. Despite its initial reputation as potential psychotherapeutical drug around the 1970s, and later as an aphrodisiac, over the last decade 2C-B has gained popularity among electronic music party goers as the replacement of choice for ecstasy (MDMA, Molly) and LSD, either alone or combined (González et al., 2013; Fernández-Calderón et al., 2017). Based on the abrupt introduction of 2-CB onto the drug market, 2C-B and any of its salts or isomers were added to Schedule II of the 1971 Convention on Psychotropic Substances by the UN Commission on Narcotic Drugs in 2001 (United Nations Office on Drugs and Crime [UNODC], 1971; de Boer and Bosman, 2004). The European Monitoring Centre for Drugs and Drug Addiction (EMCDDA) and the United Nations Office on Drugs and Crime (UNDOC) classified it as an NPS (European Monitoring Centre for Drugs and Drug Addiction [EMCDDA], 2011; United Nations Office on Drugs and Crime [UNODC], 2013). In some Latin American countries as Colombia, also 2C-B is considered an NPS due to its recent presence in the market (Colombia National Study of Psychoactive Substance Consumption, 2013).

From a pharmacological point of view, preclinical studies have demonstrated that 2C-drugs inhibit the norepinephrine (NE) and serotonin transporters (NET and SERT, respectively) with very low potency in comparison to amphetamines (Glennon et al., 1984; Vollenweider and Kometer, 2010). Regarding 2C-B, as other hallucinogenic phenethylamines, is a partial agonist of 5HT_2A_, 5HT_2B_, and 5HT_2C_ receptors (Rickli et al., 2015; Luethi et al., 2017). Other studies however have reported that may act as a 5HT_2A_ full antagonist (Villalobos et al., 2004). It elicits weak response (5–10%) in both phospholipase A2–arachidonic acid (PLA2–AA) release and phospholipase C-inositol phosphate (PLC-IP) accumulation on 5HT2A receptors (Kurrasch-Orbaugh et al., 2003; Moya et al., 2007). The metabolism of 2C-B has been studied in experimental animals and *in vivo* models (Kanamori et al., 2002, 2005; Carmo et al., 2004; Theobald et al., 2007). It is generally assumed that 2C-B is metabolized mainly by the monoamine oxidase enzymes, MAO-A and MAO-B, and, to a lesser degree, by the CYP450 system (Carmo et al., 2005; Pichini et al., 2008; Kanamori et al., 2013, 2017). Urine analysis from a 2C-B abuser have identified and quantified unchanged 2C-B and nine different metabolites suggesting that 2C-B is metabolized to an alcoholic metabolite [4-bromo-2,5-dimethoxyphenylethylalcohol (2C-B-ALC)], and a carboxylated metabolite [4-bromo-2,5-dimethoxyphenylacetic acid (2C-B-CBA) (Kanamori et al., 2013, 2017). Currently, there are not data available describing active metabolites that could contribute to overall effects, therefore, 2C-B seems the active one while the rest are inactive or nearly so.

Data concerning prevalence and patterns of use of novel psychedelics are limited. In 2013, almost half of the Global Drug Survey [GDS] (2013) respondents (2,282, 46.4%) reported lifetime use of at least one NPS. Of these, 21.7% described psychedelic phenethylamine lifetime use, with 18.4% corresponding to the 2C series, the most common being 2C-B (*n* = 291, 12.97%) (Palamar et al., 2016). In Australia, a survey among regular ecstasy users showed that 44% had used an NPS in the last 6 months, mainly 4-iodo-2,5-dimethoxyphenethylamine (2C-I, 14%) and 2C-B (8%) (Burns et al., 2014). In a cross-sectional survey carried out at music festivals among 230 research chemical users, the most frequent substance employed was 2C-B (80.0%). Among the most frequent combinations were 2C-B with MDMA (28.3%), less prevalent were 2C-B with amphetamine, LSD, ketamine, and methylone (7.4, 5.7, 3.9, and 2.6%, respectively) (González et al., 2013). The latest data from the GDS, which included a non-representative sample of 115,000 subjects, suggest an increase in the consumption of drugs with a psychedelic effect profile (including LSD analogs), representing over 50% of the total NPSs. Estimated life-time and past year use of 2C-B was 5.1 and 2.7%, respectively (Global Drug Survey [GDS], 2017). Regarding trends in the United Kingdom, psychedelic use over the last 4 years was stable at around 7% with the exception of 2016 in which it rose to 9.8% (7.7-6.3-9.8-7.1%). Globally, 6.3% of the previous 12 month 2C-drug users suffered difficult/negative experiences while under the influence of psychedelics. In Central and South America, 2C-B has become a very popular nightlife NPS (Colombia National Study of Psychoactive Substance Consumption, 2013). 2C-B is usually taken orally in powder or tablet form, in doses of 10–30 mg. Tablets typically contain 5–10 mg of the substance. An oral low dose is considered to be 5–10 mg, a medium dose 10–25 mg, and a high dose 25–40 mg (Caudevilla-Gálligo et al., 2012; Nugteren-van Lonkhuyzen et al., 2015; Papoutsis et al., 2015).

With the exception of emerging 2C-B research performed in the 1950s–1970s by Shulgin, who reported a maximum oral dose of 100 mg without apparent harm (Shulgin and Carter, 1975; Shulgin and Shulgin, 1990), limited clinical research has been conducted in humans. Current evidence about 2C-B acute effects in humans comes from intoxications collected at Poison Information Centers (Burns et al., 2014; Srisuma et al., 2015), self-reports from research chemical recreational users (questionnaires and surveys) (Caudevilla-Gálligo et al., 2012; González et al., 2013), and intoxication cases (Ambrose et al., 2010; Huang and Bai, 2011; Hondebrink et al., 2015; Liakoni et al., 2015; Caicedo et al., 2016), the clinical presentation including typical hallucinations (tactile, visual, and auditory) and neuropsychiatric symptoms (anxiety, agitation, and confusion). While a number of fatalities have been linked to other substances in the 2C-drug group none have been attributed to 2C-B alone.

We have recently published a manuscript about the acute pharmacological effects of 2C-B focused on emotions. Results showed a specific profile suggesting 2C-B classification as an entactogen drug with psychedelic properties (González et al., 2015). The purpose of the present study is to assess the acute pharmacological effects and oral fluid pharmacokinetics of 2C-B in humans.

## Materials and Methods

### Participants

Sixteen healthy volunteers were included (eight males and eight females). Subjects were recreational drug users who reported having used 2C-B at least once in their lives. Exclusion criteria were history of any serious medical or mental disorder including drug dependence (except for nicotine), use of chronic medication, and serious adverse reactions with 2C-B.

Participants were recruited by worth-of-mouth through the Association for the Study of States of Consciousness (PHI). The protocol was approved by the Local Human Research Ethical Committee (CEIC Parc de Salut Mar, Barcelona, Spain) and all the participants were informed about the purpose and procedures of the study, and signed an informed consent prior to any study-related procedure. The study was conducted in accordance with the Declaration of Helsinki. Participants received financial compensation for their participation.

### Design and Treatments

A non-controlled prospective observational study was conducted. Each subject participated in one session. They ingested a capsule that they brought to the testing site themselves, which they had obtained from an unknown source. Although no information was available about the synthesis of the drug, similar capsules tested by Energy Control, a harm reduction organization that provides a Drug Checking Service for users, showed that the capsules contained 2C-B at 95% purity with no toxic adulterants. The 2C-B pill content was previously analyzed by means of gas chromatography associated with mass spectrometry (GC/MS). The method used permits to check for most common drugs of abuse including cocaine, MDMA, LSD, amphetamine and methamphetamine, heroin, 2C-B and other phenethylamines, DMT and other tryptamines, ketamine, psilocybin, salvinorin A, natural and synthetic cannabinoids, and most of the NPSs (Caudevilla-Gálligo et al., 2012; González et al., 2015; Grifell et al., 2017; Palma-Conesa et al., 2017; Quintana et al., 2017). Participants were given a choice of three doses to choose from, 10, 15 or 20 mg 2C-B, based on their stated preference from previous experience. They chose to take a mean 2C-B dose of 15.94 ± 4.17 mg (four subjects ingested 10 mg, five subjects 15 mg, and seven subjects 20 mg).

#### Procedures

Prior to participation all subjects were trained with respect to the procedures, tests, and questionnaires employed in the study. Participants were requested to abstain from any drug use 48 hours (h) prior to the study session. Alcohol and caffeine-containing beverages were not allowed the previous 24 h (or the morning of the study session). Sessions took place on different days at the home of a member of the PHI Association. The setting included ambience music (except in the evaluation times). Subjects could read, talk, play table games during sessions and interact. They were instructed not to talk about the effects of the substance during the session. Assessments were performed at baseline (predose, immediately before 2C-B self-administration) and over 6 h after 2C-B self-administration. The experiments were conducted at the same time for all subjects, from 15:00 to 22:00 h. A light snack was ingested immediately after. Urine spot samples were collected before 2C-B administration to exclude drug use prior to the session (MDMA, amphetamines, barbiturates, benzodiazepines, cocaine, marijuana, morphine, methamphetamine, phencyclidine with Instant-View, Multipanel 10 Test Drug Screen Alfa Scientific Designs, Inc., Poway, CA, United States). 2C-B self-administration took place approximately at 16.00 h.

The sequence of procedures at each time point of the session was: vital signs, physiological effects, oral fluid collection, subjective effect scales and questionnaires.

#### Vital Signs/Physiological Effects

Systolic blood pressure (SBP), diastolic blood pressure (DBP), and heart rate (HR) were measured with an automatic Omron^®^ monitor at baseline and 1, 2, 3, 4, and 6 h after self-administration.

#### Subjective Effects

Subjective effects were recorded at baseline and 2 h after administration, at 6 h subjects were asked to report the maximum effects along 6 h (0–6 h). The Hallucinogenic Rating Scale (HRS) was completed only at 6 h post-administration.

Subjective effects of 2C-B were measured using a set of the visual analog scale (VAS), the Addiction Research Center Inventory (ARCI), the Evaluation of the Subjective Effects of Substances with Abuse Potential (VESSPA-SSE) questionnaires, and the HRS.

Visual analog scale (100 mm, from “not at all” to “extremely”) were used to rate intensity; high; good effects; bad effects; liking; changes in distances; changes in colors; changes in shapes; changes in lights; hallucinations-seeing of lights or spots; hallucinations-seeing animals, things, insects, or people; changes in hearing; hallucinations-hearings of sounds or voices; drowsiness; dizziness; confusion; fear; depression or sadness; different body feeling; unreal body feeling; different surroundings; and unreal surroundings (González et al., 2015; Papaseit et al., 2016).

The ARCI is a true/false 49-item questionnaire is a sensitive instrument for determining subjective drug effects, and consists of five subscales: PCAG (pentobarbital-chlorpromazine-alcohol, a measure of sedation), LSD (lysergic acid diethylamide group, a measure of dysphoria and somatic symptoms), MBG (morphine-benzedrine group, a measure of euphoria), BG (benzedrine group, a stimulant scale consisting mainly of items relating to intellectual efficiency and energy), and A (amphetamine, an empirically derived scale sensitive to the effects of D-amphetamine) (Haertzen, 1965; Lamas et al., 1994).

The VESSPA-SE questionnaire measures changes in subjective effects caused by a number of drugs. It includes six subscales: sedation (S), psychosomatic anxiety (ANX), changes in perception (CP), pleasure and sociability (SOC), activity and energy (ACT), and psychotic symptoms (PS) (González et al., 2015; Papaseit et al., 2016).

The HRS includes 100 items distributed in six scales: (i) somaesthesia (reflecting somatic effects including interoceptive, visceral, and tactile effects); (ii) affect (sensitive to emotional and affective responses); (iii) volition (indicating the subject’s capacity to willfully interact with his/her ‘self’ and or the environment); (iv) cognition (describing alterations in thought processes or content); (v) perception (measuring visual, auditory, gustatory, and olfactory experiences); and (vi) intensity (which reflects the strength of the overall experience) (Strassman et al., 1994; Riba et al., 2001a).

#### Oral Fluid Concentrations of 2C-B and Cortisol

Oral fluid (saliva) was collected with Salivette^®^ tubes to assess 2C-B and cortisol concentrations at baseline, 1, 2, 3, 4, 6, 16, and 24 h after administration (*n* = 8 for cortisol). Oral fluid samples were centrifuged and frozen at -20°C until analysis. 2C-B concentrations were quantified with gas chromatography–mass spectrometry (GC–MS) (González et al., 2015). Cortisol samples were analyzed with the AxSYM Cortisol Assay (Abbott Diagnostics, Abbott Park, IL, United States) which utilizes fluorescence polarization immunoassay (FPIA) according to the manufacturers’ instructions.

### Statistical Analysis

Differences with respect to baseline were calculated for vital signs (SBP, DBP, and HR) and subjective effects (VAS, ARCI, and VESSPA). Maximum effects (E_max_) and the time needed to reach maximum effects (t_max_) were also calculated for the mentioned variables. The area under the curve of the concentrations (AUC) using the trapezoidal rule were calculated for vital signs.

The AUC, the maximum concentration (C_max_) and the time needed to reach the maximum concentration (t_max_), elimination half-life (t_1/2_) and elimination constant (K_e_), from 2C-B and cortisol oral fluid concentrations over time were determined using Pharmacokinetic Functions for Microsoft Excel (Joel Usansky, Atul Desai, and Diane Tang-Liu, Department of Pharmacokinetics and Drug Metabolism, Allergan, Irvine, CA, United States).

Firstly, a two-way analysis of variance (ANOVA) test was conducted to study the influence of dose and gender in the different parameters calculated. Because the results showed only marginal statistically significant results for interactions between dose and gender, dose or gender, the analysis was rejected (nine variables showed significant results for a total number of 198 comparisons). Subsequently, the statistical analysis presented was performed without considering these factors.

E_max_ values of vital signs and cortisol were compared with baseline data using a paired samples *t*-test. Furthermore, a detailed comparison between different time points was performed by means of a one-way repeated measures ANOVA, with time condition as factor. When the time condition was statistically significant, a Dunnett *post hoc* test was performed to compare the different time points with baseline.

For subjective effects, a one-way repeated measures ANOVA was performed with time condition as factor (baseline, 2 and 6 h). When ANOVA has been statistically significant a Dunnett *post hoc* test was performed to compare 2 and 6 h with baseline. No comparison between 2 and 6 h (maximum effects from 0 to 6 h) was performed because both measures although obtained in different time points are an approximation of the same parameter (E_max_).

Statistical analysis was performed using PAWS Statistics version 18 (SPSS, Inc., Chicago, IL, United States). A value of *p* < 0.05 was considered statistically significant and it was adjusted for the multiple comparisons.

## Results

### Participants

A total of 16 healthy subjects participated in the study (eight males and eight females). They had a mean age of 33.25 ± 3.71 years (range: 27–39), weighed 63.81 ± 11.59 kg (range: 44–84), and their mean body mass index (BMI) was 21.69 ± 2.49 kg/m^2^ (range: 18.6–27). The mean 2C-B weight-adjusted dose was 0.27 ± 0.09 mg/kg (range: 0.12–0.45). They reported an average previous 2C-B use of 3 (range: 1–20) times during their lifetime. All volunteers had recreational experience with MDMA, amphetamines, hallucinogens, cocaine, and cannabis. 12 were current tobacco smokers (range: 5–30 cigarettes/day) and all of them consumed alcohol (mean: 1 unit/day). Baseline drug urine tests were negative.

### Vital Signs/Physiological Effects

**FIGURE 1:**
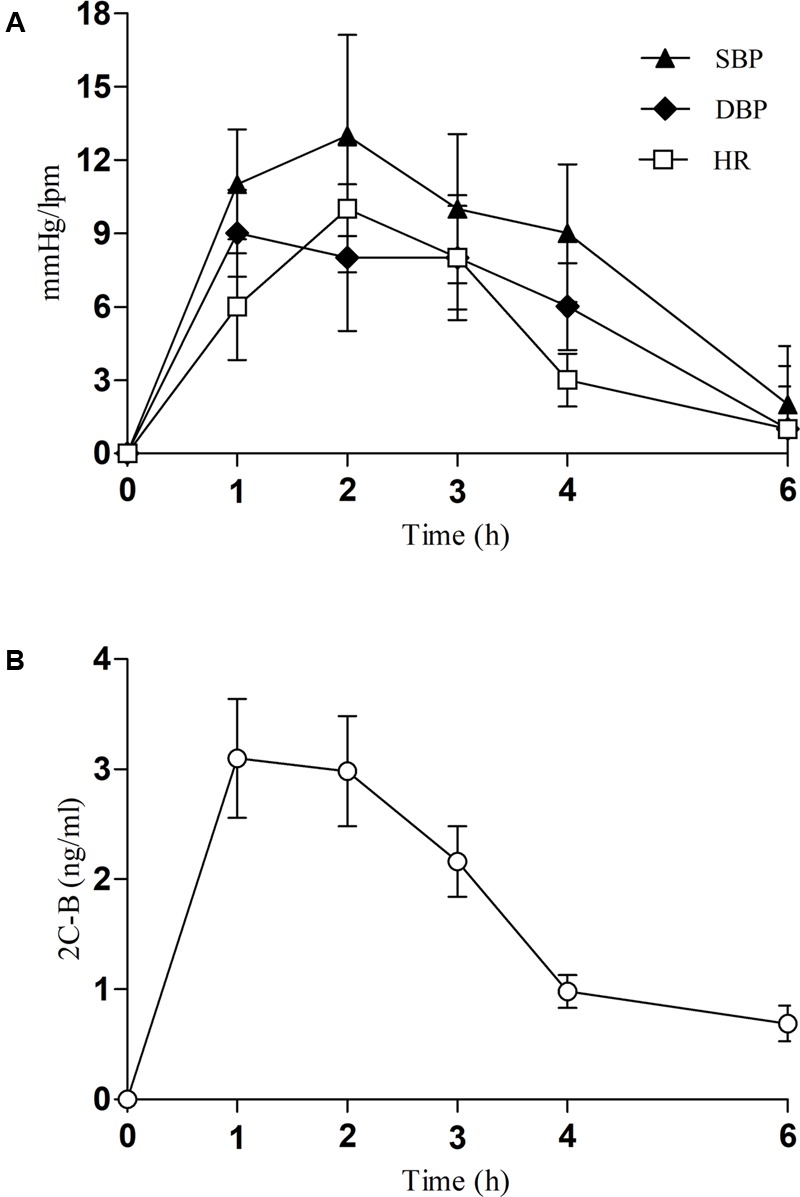
**(A)** Time course of changes from baseline for systolic and diastolic blood pressure (SBP, DBP, mmHg) and heart rate (HR, bpm, beats per minute) (*n* = 16, mean, standard error). **(B)** Time course of 2C-B concentrations in oral fluid (*n* = 16, mean, standard error).

Changes in vital signs/physiological outcomes are shown in **Table 1** and **Figure 1A**. 2C-B produced an increase in SBP, DBP, and HR. Maximum effects (E_max_) were +19 mmHg, +13 mmHg, and +13 bpm, respectively. Compared to baseline values, statistically significant differences were detected for SBP from 1 to 4 h and from 1 to 3 h for DBP and HR. For both SBP and HR, median t_max_ values ranged from 1 to 4 h whereas for DBP t_max_ ranged from 1 to 2 h. Time course of changes were similar to oral fluid concentration of 2C-B (see below, **Figure 1B**).

**Table 1:** Summary of result on the systolic and diastolic blood pressure (SBP, DBP) and heart rate (HR) (*n* = 16) observed after self-administration of 2C-B.

Vital signs/physiological effects	Parameter	ANOVA/T student		Comparison to baseline	Mean ±*SD*
		F/T	*p*-Value	Dunett’s test	
SBP	E_max_	5.740	<0.001		19.25 ± 13.41
	T-C (df = 1,75)	7.119	<0.001	1, 2, 3, 4 h	
DBP	E_max_	5.910	<0.001		13.13 ± 8.88
	T-C (df = 1,75)	6.460	<0.001	1, 2, 3 h	
HR	E_max_	6.060	<0.001		12.63 ± 8.33
	T-C (df = 1,75)	6.813	<0.001	1, 2, 3 h	

*E_*max*_ = peak effects 0–6 h (differences from baseline) measured by mmHg (SBP, DBP), bpm (HR). T-C = time course from 0 to 6 h. For T-C an ANOVA and a *post hoc* Dunnett’s test for multiple comparisons was used.*

### Subjective Effects

2C-B produced robust changes in most subjective effects measured by VAS, ARCI, and VESSPA-SEE. **Table 2** shows the results for the different subscales of the questionnaires.

**Table 2:** Summary of result on subjective effects (*n* = 16) observed after self-administration of 2C-B.

Subjective effects	Time (h)	Mean ± *SD*	ANOVA		Comparison to baseline
			*F*	*p*-Value	Dunett’s test
**Visual analog scale (VAS)**					
Intensity	2	45.13 ± 21.07	64.622	<0.001	<0.001
	6	59.68 ± 20.18			<0.001
High	2	69.44 ± 17.36	189.515	<0.001	<0.001
	6	67.75 ± 17.40			<0.001
Good effects	2	68.25 ± 19.26	176.377	<0.001	<0.001
	6	70.00 ± 15.43			<0.001
Bad effects	2	3.63 ± 5.23	3.023	0.064	
	6	10.50 ± 21.33			
Liking	2	78.00 ± 19.77	185.113	<0.001	<0.001
	6	76.69 ± 23.41			<0.001
Changes in distances	2	28.19 ± 26.72	14.252	<0.001	<0.001
	6	25.38 ± 23.10			<0.001
Changes in colors	2	37.50 ± 29.68	20.220	<0.001	<0.001
	6	34.44 ± 27.75			<0.001
Changes in shapes	2	36.94 ± 32.39	10.567	<0.001	<0.001
	6	32.25 ± 29.66			<0.001
Changes in lights	2	42.19 ± 31.12	18.549	<0.001	<0.001
	6	35.63 ± 26.57			<0.001
Hallucinations-seeing of lights or spots	2	13.50 ± 21.90	6.138	0.006	0.036
	6	18.50 ± 28.27			0.004
Hallucinations-seeing animals, things, insects, or people	2	6.75 ± 21.61	1.609	0.217	
	6	5.37 ± 15.55			
Changes in hearing	2	9.25 ± 15.91	3.883	0.032	0.020
	6	6.38 ± 6.90			0.125
Hallucinations-hearings of sounds or voices	2	3.25 ± 7.90	2.021	0.150	
	6	1.69 ± 3.18			
Drowsiness	2	13.19 ± 20.37	5.918	0.007	0.027
	6	16.50 ± 22.66			0.005
Dizziness	2	8.75 ± 21.29	2.060	0.145	
	6	7.00 ± 16.12			
Confusion	2	6.81 ± 9.11	4.115	0.026	0.103
	6	9.63 ± 17.19			0.017
Fear	2	0.69 ± 1.54	1.086	0.350	
	6	2.25 ± 7.44			
Depression or sadness	2	1.38 ± 2.92	1.163	0.326	
	6	3.88 ± 13.66			
Different body feeling	2	60.38 ± 21.69	53.946	<0.001	<0.001
	6	56.50 ± 26.34			<0.001
Unreal body feeling	2	13.06 ± 21.66	3.533	0.042	0.024
	6	7.19 ± 13.52			0.262
Different surroundings	2	29.19 ± 28.51	12.102	<0.001	<0.001
	6	19.94 ± 22.97			0.005
Unreal surroundings	2	3.19 ± 5.65	3.476	0.044	0.027
	6	2.13 ± 3.80			0.165
**ARCI questionnaire**					
PCAG	2	4.19 ± 2.86	0.121	0.887	
	6	4.31 ± 3.14			
MBG	2	7.13 ± 3.65	45.812	<0.001	<0.001
	6	6.19 ± 3.60			<0.001
LSD	2	6.44 ± 2.28	7.580	0.002	0.002
	6	5.94 ± 2.95			0.012
BG	2	5.50 ± 2.39	5.929	0.007	0.009
	6	5.44 ± 2.06			0.013
A	2	4.88 ± 1.86	86.834	<0.001	<0.001
	6	4.06 ± 1.44			<0.001
*VESSPA-SEE questionnaire*					
Sedation (S)	2	5.63 ± 4.43	14.787	<0.001	<0.001
	6	5.06 ± 6.02			<0.001
Psychosomatic anxiety (ANX)	2	3.19 ± 2.51	20.556	<0.001	<0.001
	6	2.81 ± 2.48			<0.001
Changes in perception (SP)	2	3.69 ± 4.39	10.099	<0.001	0.001
	6	3.56 ± 4.03			0.001
Pleasure and sociability (SOC)	2	12.13 ± 5.26	59.280	<0.001	<0.001
	6	9.88 ± 5.11			<0.001
Activity and energy (ACT)	2	6.88 ± 4.18	20.797	<0.001	<0.001
	6	6.06 ± 4.73			<0.001
Psychotic symptoms (PS)	2	0.81 ± 1.05	7.291	0.003	0.003
	6	0.75 ± 0.93			0.007

*Subjective effects measured by mm (VAS) and score (ARCI and VESSPA-SEE questionnaires). Time 2 h (effect at 2 h after administration). Time 6 h (maximum effects from 0 to 6 h).*

2C-B self-administration increased the score in all the outcomes measured with VAS. The highest scores (a difference of >50 mm from baseline) were obtained for intensity, high, good effects, and different body feeling scales. Differences of >25 mm from baseline were obtained for changes in distances, colors, shapes, and light scales while moderate (<15 mm) and small changes (<10 mm) were found in the scales measuring hallucinations, changes in hearing, and unreal surroundings. In comparison to baseline, statistical significant changes were detected for all VAS scales with the exception of bad effects, hallucinations-seeing animals, things, insects, or people, hallucinations-hearings of sounds or voices, dizziness, fear, and depression or sadness. In fact only five of the subjects described clear hallucinogenic effects in VAS.

Regarding the effects measured with the ARCI questionnaire, after 2C-B administration significant changes in all ARCI subscales were observed, expect for PCAG (sedation). The most marked increases compared to baseline were found for MBG (euphoria) and A (amphetamine) subscales. Modest increases were detected for LSD (dysphoria and somatic symptoms) and BG (intellectual efficiency and energy) subscales.

In relation to the VESSPA-SP questionnaire, 2C-B induced significant increases compared to baseline in all subscales. The main changes were observed in SOC (pleasure and sociability), ACT (activity and energy), and S (sedation) subscales.

For several subjective outcomes, mean peak effects reported at 6 h (summary effects of the 0–6 h) were slightly lower than scores obtained at 2 h after administration (see **Table 2**). Nevertheless, when statistical differences from baseline were observed at 2 h, the same occurred for 6 h (maximum effects from 0 to 6 h). These differences between 2 and 6 h could be explained due to memory bias.

**FIGURE 2:**
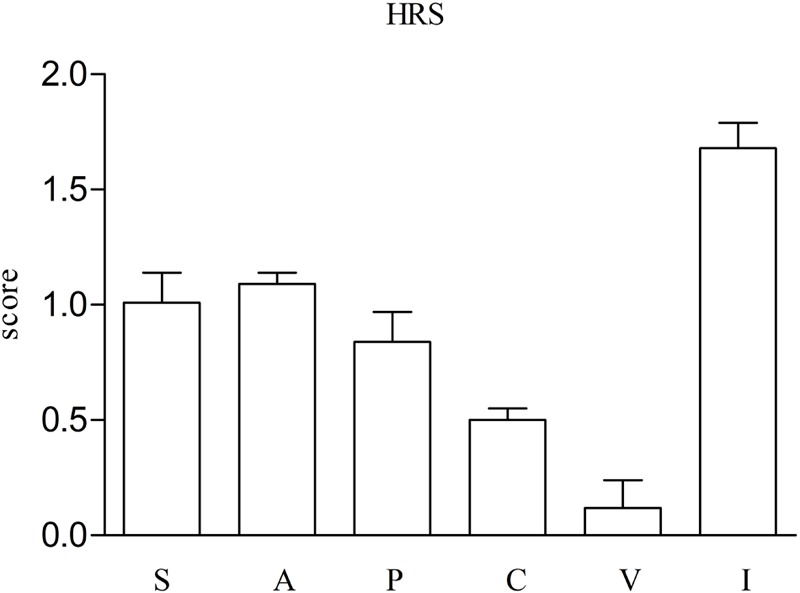
Scores on The Hallucinogenic Rating Scale (HRS) questionnaire subscales (*n* = 16, mean, standard error). Scores represent the maximum effects along 6 h after self-administration of 2C-B. S: somaesthesia; A: affect; P: perception, C: cognition; V: volition, I: intensity.

With respect to the HRS, the highest scores were obtained for intensity, volition and affect subscales (see **Figure 2**).

### Oral Fluid Concentrations

2C-B oral fluid concentrations increased quickly after 2C-B ingestion, reaching a peak (t_max_) 1 h after self-administration (**Figure 1B**). Concentrations decreased rapidly from 2 to 6 h after ingestion and could be detected in oral fluid up to 24 h in half of the volunteers. C_max_ reached was 4.19 ± 1.86 ng/ml and AUC from 0 to 24 h was 19.54 ± 4.72 ng × h/ml. 2C-B elimination half-life (t_1/2_) in oral fluid was 2.48 ± 3.20 h. Data from one volunteer were excluded due to outlier concentrations (an analytical error was suspected). As mentioned previously, both concentrations and vital signs time course were similar (**Figures 1A,B**).

Cortisol concentrations were measured in a subset of eight volunteers. Cortisol baseline concentrations were 0.64 ± 0.46 μg/dl. After 2C-B administration concentrations reached a C_max_ of 1.13 ± 0.23 μg/dl at 3 h (t_max_) (not statistically significant). From 3 to 4 h concentrations abruptly decreased returning slowly to baseline 16 h after administration.

## Discussion

This study assessed the acute pharmacological effects of 2C-B in a non-controlled setting. The main finding is that 2C-B produces a constellation of psychedelic-psychostimulant like effects, a profile consistent with previous human data (Caudevilla-Gálligo et al., 2012; González et al., 2015). Our research provides unique results regarding 2C-B concentrations in oral fluid and cortisol.

Additionally, results show that the self-administration of 2C-B at the narrow dose range studied (10–20 mg) in healthy experienced users in a non-medical setting is relatively safe (González et al., 2015). In contrast to our previous publication (González et al., 2015), that focused on the effects of 20 mg on emotions, the present study included a more intensive evaluation of vital signs, and a more complete collection of oral fluid. These evaluations provided a picture of the time-course of the effects of 2C-B on physiological measures and permitted a comparison between the time-course of the effects and the concentrations of 2C-B in oral fluid. Again, our findings concur with the limited number of cases reporting severe acute toxicity related to 2C-B use.

In a non-controlled setting, the profile of physiological effects produced by 2C-B is characterized by moderate increases of blood pressure (SBP and DBP) and HR, but lower than those of MDMA, amphetamines, and related compounds administered in controlled conditions (Mas et al., 1999; Papaseit et al., 2016). The onset of cardiovascular effects occurred at 1 h assessment and maintained over a long-lasting period (4 h). At 6 h values returned toward pre-drug self-administration.

The subjective effect of 2C-B in this study consists of mixed euphoric, well-being reactions and alterations in mental functions closely related to psychostimulants such as MDMA, amphetamine, and mephedrone, and psychedelics such as ayahuasca, salvinorin A, and Salvia Divinorum. Globally, subjects under 2C-B effects reported euphoria, activation and a psychedelic experience consisting of a temporary altered state of consciousness. Mood changes were more prominent that perceptual changes. Specifically, the mean VAS ratings of liking, good effects, and high (up to 78% of maximum possible VAS scores) were even greater than those determined in experimental conditions for MDMA and other related psychostimulants (Mas et al., 1999; Papaseit et al., 2016). In relation to the MBG subscale, considered a measure of drug-induced euphoria, 2C-B induced high scores which, when regarding to psychedelics, are indicative of subjective feelings of well-being and confidence. As previously postulated for other psychedelics, it is possible that euphoria may also be an essential component of the psychedelic experience after 2C-B use (Bouso et al., 2016). Interesting, 2C-B also resulted in increases in the LSD subscale and somatic VAS scales (drowsiness, dizziness, confusion). Despite the coexistence of dysphoric-somatic effects, the induced well-being and pleasant effects were clearly more important as reflected by the increases in rating scores. In contrast, fear and visual hallucinations were not experienced, and sedation was unremarkable. Is it noteworthy that alteration in perception ranged from changes in perceptions to hallucinations, although the latter were only experienced by 2 (hearing of sounds or voices, 10–36 mm score) and 3 subjects (seeing of lights or spots, 10–87 mm score). Such results differ from other psychedelics probably due to the relatively low-moderate doses self-administered in this study.

The HRS has been previously used to measure the hallucinatory effects of *N,N*-dimethyltryptamine (Strassman et al., 1994), ayahuasca (Riba et al., 2001b, 2004), psilocybin (Griffiths et al., 2006), salvinorin A (Johnson et al., 2011), and MDMA (Tancer and Johanson, 2007) among others. Our results showed the highest scores for intensity, volition and affect subscales. The scores for some subscales in the present study were lower (somaesthesia, perception, cognition, and intensity) or similar (affect and volition) than described in experimental studies administering other psychedelics as N,N-dimethyltryptamine (Strassman et al., 1994), ayahuasca (Riba et al., 2001b, 2004), psilocybin (Griffiths et al., 2006), and salvinorin A (Johnson et al., 2011). Interestingly, the present study found that the HRS scores were similar to those 2C-B ratings reported by recreational psychedelic users. Similar changes were observed in volition subscale whilst, in comparison, lower scores were obtained for intensity, somaesthesia, perception, affect, and cognition subscales (Caudevilla-Gálligo et al., 2012).

The psychedelic effects produced by 2C-B are varied and include somatic symptoms (dizziness, drowsiness, and confusion), perceptual symptoms (changes in distances, colors, shapes, lights, different body feelings, different surroundings, unreal body feelings, and unreal surroundings) and visual hallucinations Subjects under 2C-B effects reported a psychedelic experience consisting of euphoria and the activation, but not experience, of typical hallucinations. Results were similar to those described in previous works and surveys, all symptoms were resolved by 6 h (Caudevilla-Gálligo et al., 2012; Nugteren-van Lonkhuyzen et al., 2015).

In a similar manner to other NSPs and some psychedelics, the human pharmacokinetics of 2C-B has not yet been fully resolved. Analysis of oral fluid samples by LC–MS/MS concentrations ranged from 1.43 to 7.73 ng/ml, with an average peak concentration of 4.19 ng/ml observed between 1 and 3 h after administration. However, results of 2C-B pharmacokinetics indicate that 2C-B can be detected in oral fluid at very low concentrations up to 16–24 h after self-administration. Unfortunately, the interpretation of 2C-B concentrations in oral fluid is extremely difficult without data from plasma (not performed in this study neither in any study involving humans). By contrast, collection of oral fluid is easy and non-invasive, and was the method selected to obtain pharmacokinetic data in this study. Anecdotally, in two subjects involved in a road accident, 2C-B has been identified in blood at concentrations of 1.6 and 14 ng/ml together with amphetamine (Busardò et al., 2017).

After 2C-B administration, the increase in cortisol concentration was very small and no statistically significant. For other serotonergic psychedelic and psychedelic-like drugs including MDMA, ayahuasca, and psilocybin (Mas et al., 1999; Hasler et al., 2004; Dos Santos et al., 2011; Hysek et al., 2014; Seibert et al., 2014; Farré et al., 2015) a market increases in plasma cortisol concentrations have been detected.

Our work has several limitations which are mainly associated with its naturalistic observational design. Firstly, the study was open label without a lack of control/placebo, therefore an expectancy bias cannot be discarded. Secondly, data were obtained from a small sample of experienced psychedelic drug users, including both genders. Thirdly, a limited dose range was evaluated, the dose was selected by the participants according to their preferences and was relatively low-moderate (10–20 mg). Higher doses (>25 mg) are reported to cause unpleasant hallucinations and sympathomimetic effects such as tachycardia, hypertension and hyperthermia (Huang and Bai, 2011). Therefore, the observed acute effects may be useful in a similar subpopulation of polydrug-users but should be extrapolated with caution to the general population. Fourth, the exclusive reliance on subjective effects with few objective measures. Fifth, the setting used could influence the effects reported by participants. In addition, our findings may not apply to other routes of 2C-B administration. Finally, the effects obtained at 6 h are not a substitute for the assessment of the subjective effects in real peak effect time, ideally performed with a clinical trial design. Nonetheless, this study investigated the acute subjective effects using validated questionnaires with proven sensitivity for discriminating subjective effects, and validated analytic techniques for 2-CB and cortisol determinations.

## Conclusion

The results presented in this work constitute a preliminary approach to the acute physiological and subjective effects and pharmacokinetics of 2C-B. According to these preliminary results, oral fluid could be a suitable biologic matrix to detect 2C-B acute use. They suggest that oral 2C-B self-administration in experienced drug users, in a non-controlled setting, induces a constellation of psychedelic/psychostimulant like effects commonly associated with drugs that have a greater influence on serotonin action.

Further experimental research under controlled conditions is needed to compare human pharmacology of 2C-B with other classical drugs.

## Author Contributions

MF, MT, MV, and DG: conceptualized the study design; MF and DG: collected the data; RdlT and MP: analyzed the oral fluid; MV: analyzed the 2C-B contents; MF, EP, CP-M, DG, RdlT, MV, and MT: written the manuscript.

## Conflict of Interest Statement

The authors declare that the research was conducted in the absence of any commercial or financial relationships that could be construed as a potential conflict of interest.
